# Nosocomial Co-Transmission of Avian Influenza A(H7N9) and A(H1N1)pdm09 Viruses between 2 Patients with Hematologic Disorders

**DOI:** 10.3201/eid2204.151561

**Published:** 2016-04

**Authors:** Huazhong Chen, Shelan Liu, Jun Liu, Chengliang Chai, Haiyan Mao, Zhao Yu, Yuming Tang, Geqin Zhu, Haixiao X. Chen, Chengchu Zhu, Hui Shao, Shuguang Tan, Qianli Wang, Yuhai Bi, Zhen Zou, Guang Liu, Tao Jin, Chengyu Jiang, George F. Gao, Malik Peiris, Hongjie Yu, Enfu Chen

**Affiliations:** Taizhou Hospital, Wenzhou Medical College, Taizhou, China (H. Chen, H.X. Chen, C. Zhu, H. Shao);; Zhejiang Provincial Center for Disease Control and Prevention, Hangzhou, China (S. Liu, C. Chai, H. Mao, Z. Yu, E. Chen);; National Institute for Viral Disease Control and Prevention, Chinese Center for Disease Control and Prevention, Beijing, China (J. Liu, G.F. Gao);; Linhai District Center for Disease Control and Prevention, Taizhou (Y. Tang);; Jiaojiang District Center for Disease Control and Prevention, Taizhou (G. Zhu);; Institute of Microbiology, Chinese Academy of Sciences, Beijing (S. Tan, Q. Wang, Y. Bi, G.F. Gao);; Chinese Academy of Medical Sciences, Beijing (Z. Zou, C. Jiang);; University of Chinese Academy of Sciences, Beijing (G. Liu);; BGI-Shenzhen, Shenzhen, China (G. Liu, T. Jin);; University of Hong Kong, Hong Kong, China (M. Peiris);; Division of Infectious Disease, Key Laboratory of Surveillance and Early Warning on Infectious Disease, Chinese Center for Disease Control and Prevention, Beijing (H. Yu)

**Keywords:** H7N9, cluster, human-to-human transmission, nosocomial transmission, immunocompromised, co-infection, pandemic, A(H1N1)pdm09, A(H1N1)pdm2009, pH1N1, H1N1, influenza, viruses, hematologic disorders, chronic lymphocytic leukemia, polycythemia vera, zoonoses

## Abstract

A nosocomial cluster induced by co-infections with avian influenza A(H7N9) and A(H1N1)pdm09 (pH1N1) viruses occurred in 2 patients at a hospital in Zhejiang Province, China, in January 2014. The index case-patient was a 57-year-old man with chronic lymphocytic leukemia who had been occupationally exposed to poultry. He had co-infection with H7N9 and pH1N1 viruses. A 71-year-old man with polycythemia vera who was in the same ward as the index case-patient for 6 days acquired infection with H7N9 and pH1N1 viruses. The incubation period for the second case-patient was estimated to be <4 days. Both case-patients died of multiple organ failure. Virus genetic sequences from the 2 case-patients were identical. Of 103 close contacts, none had acute respiratory symptoms; all were negative for H7N9 virus. Serum samples from both case-patients demonstrated strong proinflammatory cytokine secretion but incompetent protective immune responses. These findings strongly suggest limited nosocomial co-transmission of H7N9 and pH1N1 viruses from 1 immunocompromised patient to another.

## Abstract 2

Transmission of these viruses was limited to 2 immunocompromised patients in the same ward.

As of January 4, 2016, a novel avian influenza A virus, A(H7N9), first identified in China in March 2013 (*1*), had caused 676 laboratory-confirmed cases of influenza in humans and 275 influenza-associated deaths in mainland China (Chinese Center for Disease Control and Prevention, unpub. data). Most H7N9 virus infections have been acquired through exposure to live poultry markets (LPMs) in urban settings (*2*) and have been sporadic, but a few have occurred in clusters of >2 epidemiologically linked cases (*3*). Human-to-human transmission is difficult to prove because exposure to a common source often cannot be excluded. Nosocomial transmission of H7N9 virus has recently been reported in China’s Zhejiang Province (*4*). We investigated possible nosocomial co-transmission of H7N9 and influenza A(H1N1)pdm09 (pH1N1) viruses between 2 immunocompromised patients in Zhejiang Province.

## Methods

### Patients and Samples Collection

China’s national surveillance system for influenza-like illness (ILI), severe acute respiratory illness, and pneumonia of unexplained origin indicated a cluster of 2 H7N9 virus infections occurring in the same ward at Taizhou Hospital in Zhejiang Province, China, during January 10–15, 2014. We collected throat swabs and serum from the 2 affected case-patients for virologic, serologic, and cytokine studies. We collected specimens from the upper respiratory tract by using pharyngeal swabs or from the lower tract by using bronchoalveolar lavage. These specimens were collected on January 19 and 21 from the index case-patient and the second case-patient, respectively.

### Controls

We retrieved stored serum samples from 21 other H7N9 virus–infected patients (mean age 60 years, range 44–76 years) from Zhejiang Province (mean number of days from onset 6 days, range 2–10 days). Serum samples from 6 healthy volunteers were collected for use as controls.

### Investigation of Contacts

Close contacts were defined as described (*1*); all healthcare workers, patients sharing the same ward, and those patients’ family members were included. Close contacts were placed under daily active surveillance for 7 days. After written informed consent was obtained, a structured questionnaire was used to gather information on demographic characteristics, exposure history, and clinical outcome. Throat swab samples for H7N9 virus testing were taken from all close contacts during the observation period, and convalescent-phase serum samples were collected 3–4 weeks after the last exposure to a patient with H7N9 virus infection.

### Laboratory Methods

Specific real-time reverse transcription PCR (rRT-PCR) assays for seasonal influenza viruses (H1, H3, and B) and avian influenza viruses (H5N1 and H7N9) were conducted as described (*5*). All specimens found to be positive for virus RNA were inoculated into MDCK cell cultures for virus isolation (*2*). Virus genetic sequences were obtained directly from clinical specimens or from virus isolates by using an MiSeq desktop sequencer (Illumina, Inc., San Diego, CA, USA) as described (*6*–*8*). We designed 3 different controls to verify results: the H7N9 RNA negative and positive controls and an MDCK cell control.

Microneutralization (MN) and hemagglutination inhibition (HI) assays were performed by using A/Anhui/1/2013(H7N9) virus antigen in accordance with World Health Organization protocols (*9*). A titer of >1:40 was defined as seropositive.

Concentrations of cytokines and chemokines in serum were measured by using the Bio-PlexPro Human Cytokine Array 27-Plex Group I and 21-Plex Group II (Bio-Rad, Hercules, CA, USA). Raw data were analyzed by using xPONENT (Merck Millipore, Darmstadt, Germany).

### Statistical Analyses

A value of 0.1 pg/mL for cytokine level was assumed for statistical purposes in cases in which the concentration was undetectable. The 95% CIs for the cytokine levels of healthy controls and the control patients with H7N9 virus infection were generated by using SPSS software version 17.0 (SPSS Inc., Chicago, IL, USA).

### Ethics

This research was determined by the China’s National Health and Family Planning Commission to be part of a continuing public health outbreak investigation and therefore exempt from institutional review board assessment. The protocol for collection of epidemiologic data and serologic testing of close contacts was approved by the institutional review board of the Zhejiang Provincial Center for Disease Control and Prevention. Written informed consent was obtained from all contacts and controls who participated.

## Results

### Clinical Features of the 2 Confirmed Cases

**Figure 1:**
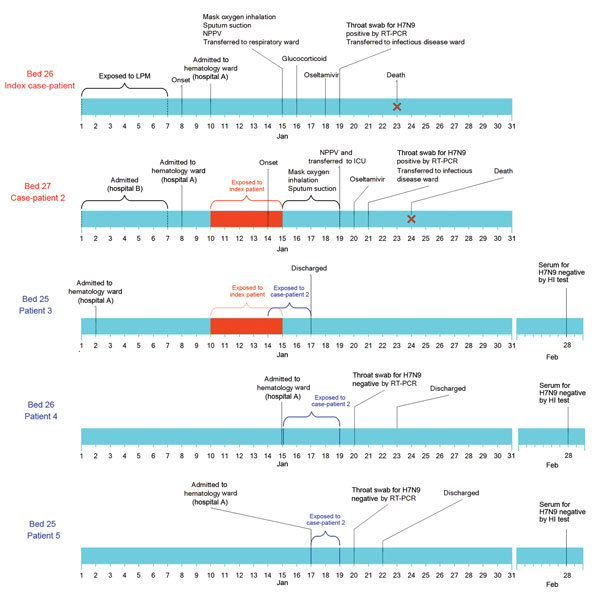
Timeline of pertinent exposures, dates of illness onset, and virologic findings for 2 patients (index case-patient and case-patient 2) who were co-infected with avian influenza A(H7N9) and A(H1N1)pdm09, and 3 non–H7N9-infected patients who shared the same hematology ward, Taizhou Hospital (hospital A), Zhejiang Province, China, January 10–15, 2014. Orange box indicates the period when patients 2–5 were exposed to the index case-patient. Blue line indicates that the period when the 3 non–H7N9-infected patients (3–5) were exposed to case-patient 2. Index case-patient was a 57-year-old man with confirmed co-infection with H7N9 and pH1N1 viruses. Case-patient 2 was a 71-year-old-man also with confirmed co-infection with H7N9 and pH1N1 viruses. Patient 3 was a 78-year-old man with chronic B lymphoma cell leukemia. Patient 4 was a 50-year-old man with acute myeloid leukemia. Patient 5 was a 61-year-old man with macroglobulinemia. H7N9, avian influenza A(H7N9) virus; HI, hemagglutination inhibition; ICU, intensive care unit; LPM, live bird market; NPPV, noninvasive positive pressure ventilation; pH1N1, influenza A(H1N1)pdm09 virus; rRT-PCR, real-time reverse transcription PCR.

**Figure 2:**
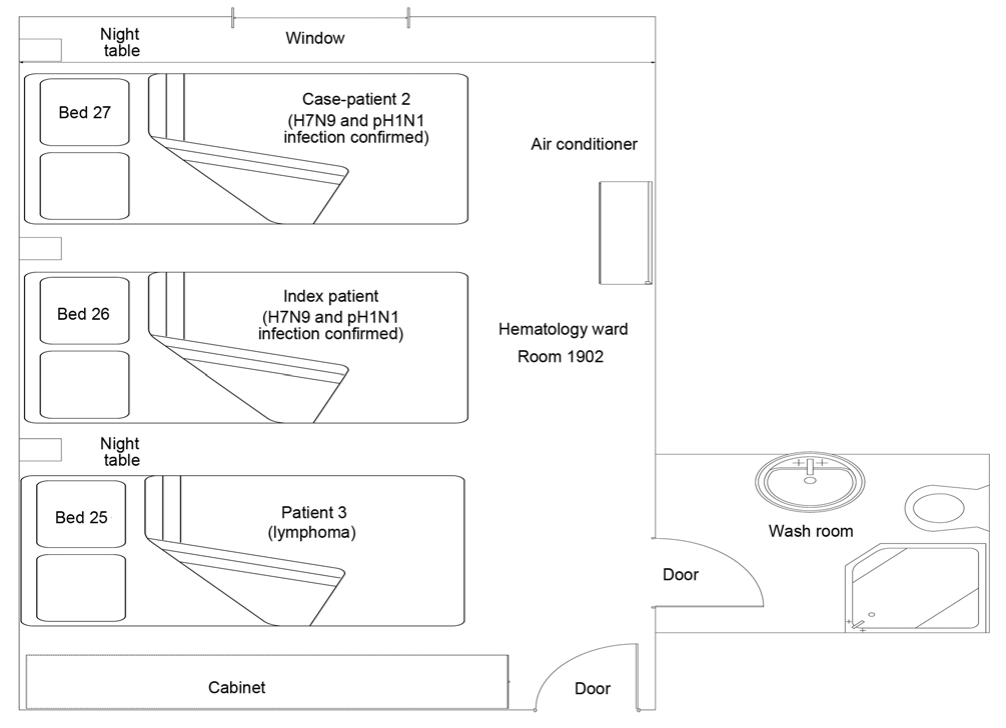
Schematic floor plan of the hematology ward where 2 case-patients with confirmed avian influenza A(H7N9) and A(H1N1)pdm09 virus co-infection and 1 non–H7N9-infected patient stayed, Taizhou Hospital, Zhejiang Province, China, January 10–15, 2014. The room was 22.4 m^2^ (6.4 m × 3.5 m) in floor area, with 1 door (30 cm × 40 cm) and 1 window (105 cm × 20 cm), and 0.6 m of space separated the beds of the patients. H7N9, avian influenza A(H7N9) virus; pH1N1, influenza A(H1N1)pdm09 virus.

The index case-patient was a 57-year-old man with untreated, Rai stage IIIA chronic lymphocytic leukemia, which manifested itself as absolute lymphocytosis, anemia, and cervical lymphadenopathy but was otherwise asymptomatic. On January 8, 2014, the patient experienced onset of fever, chills, and mild cough with sputum (Figure 1). He had not received a seasonal influenza vaccination. Two days later, he was admitted to the hematology ward at Taizhou Hospital (bed 26) because of continued fever (Figure 2; Technical Appendix Figures 1 and 2). Chest radiography performed 4 days after disease onset indicated consolidation of the left lower lung (Technical Appendix Figure 3). On day 7 of illness, the patient experienced rapid respiratory deterioration that required mechanical ventilation. He was transferred to the intensive care unit (ICU) of the respiratory ward (bed 3) (Technical Appendix Figure 4). Oral oseltamivir (75 mg twice a day) was started on day 10 of illness. A throat swab sample collected on day 11 of illness was positive for H7N9 and pH1N1 viruses. The patient was then moved to the department of infectious diseases (bed 10) for isolation (Technical Appendix Figure 5). On day 10 of illness, the geometric mean titer of the H7-specific antibody from MN was 63.5. On day 15 of illness, the patient died of complications, including acute respiratory distress syndrome, septic shock, and multiorgan failure (Figure 1; Table 1).

**Table 1:** Selected characteristics of 5 patients who shared the same hematology ward at Taizhou Hospital, Zhejiang Province, China, January 2014*

Characteristic	Index case-patient	Case-patient 2	Patient 3	Patient 4	Patient 5
Demographics and medical history					
Age, y	57	71	78	50	61
Sex	M	M	M	M	M
Type of residence	Rural	Urban	Urban	Rural	Rural
Occupation	Farmer	Retiree	Retiree	Farmer	Farmer
History of smoking	No	Yes	No	No	No
History of alcohol use	No	No	No	No	No
Obesity	No	No	No	No	No
Underlying conditions	Chronic lymphocytic leukemia; hernia operation	Polycythemia vera; cerebral infarction; diabetes	B cell lymphocytic leukemia; hypertension	Acute myeloid leukemia (M2a)	Macroglobulinemia; gout; kidney stone
Chronic drug use	No	Yes	Yes	Yes	Yes
No. family members	5	3	6	4	5
Exposure history					
Birds, other animals at home	Yes	No	No	Yes	No
Visited an LPM	Yes	No	No	Yes	No
Exposure to a febrile person	No	Yes	Yes	Yes	Yes
Duration of exposure, d (source)	—	5 (index patient)	5 (index patient); 11 (patient 2)	4 (patient 2)	3 (patient 2)
Specimen collection date (type)	Jan 19 (throat swab and serum)	Jan 21 (throat swab and serum)	Jan 26 (Serum)	Jan 20 (throat swab); Jan 26 (serum)	Jan 20 (throat swab); Jan 26 (serum)
Diagnostic method	Virus isolation; rRT-PCR; HI	Virus isolation; rRT-PCR;HI	HI	rRT-PCR;HI	rRT-PCR;HI
Date of confirmation	Jan 19	Jan 21	NA (negative)	NA (negative)	NA (negative)
Laboratory results	H7N9 and pH1N1 positive	H7N9 and pH1N1 positive	Negative	Negative	Negative
Clinical features					
Exposure to onset, d	1	4	NA	NA	NA
Onset to admission, d	2	6	NA	NA	NA
Onset to ICU, d	7	5	NA	NA	NA
Onset to antiviral drugs, d	10	6	NA	NA	NA
Onset to death, d	15	10	NA	NA	NA
Days in hospital	13	16	23	9	5
Date of outcome	Jan 23	Jan 24	Jan 17	Jan 23	Jan 22
Outcome	Died	Died	Survived	Survived	Survived

*H7N9, avian influenza A(H7N9) virus; HI, hemagglutination inhibition; ICU, intensive care unit; LPM, live poultry market; NA, not available; pH1N1, influenza A(H1N1)pdm09 virus; rRT-PCR, real-time reverse transcription PCR.

The second case-patient was a 71-year-old man with diabetes who had been undergoing insulin therapy for 20 years. He had received no vaccination against seasonal influenza. He had polycythemia vera that had been diagnosed 5 years before and was undergoing cytoreductive therapy with interferon and hydroxyurea. On December 30, 2013, he was admitted to a hospital in Jiaojiang District (also located in Taizhou) for mild fever (37.8°C) that was compatible with adverse effects from interferon therapy (Figure 1). The fever resolved by January 5, 2014. On January 8, the patient was transferred to the hematology ward (bed 27, next to the index case-patient) in Taizhou Hospital to optimize clinical management of his polycythemia vera (Figure 2). He remained afebrile until January 14, when he experienced a high fever (39.7°C) and productive cough. One day later, the patient received inhalation and parenteral methylprednisolone therapy for asthma. On January 19, shortness of breath developed, and he was moved to the hospital ICU, where tracheal intubation and mechanical ventilation were initiated (Technical Appendix Figure 6). The next day, oseltamivir treatment (75 mg 2×/d) was initiated, and throat swab samples collected for virology testing were positive for influenza H7N9 and pH1N1 viruses. The patient was transferred to the Infectious Disease Department on January 21 (Technical Appendix Figure 7). The geometric mean titers obtained through H7N9 virus MN on day 9 of illness was 113.1. The patient died on day 10 of acute respiratory distress syndrome and multiple organ failure (Figure 1; Table 1).

### Epidemiologic Links and Exposure History

The index case-patient lived with 4 family members in a 3-floor house in Linhai District. The second case-patient lived with 2 family members in an apartment in Jiaojiang District and had not come into contact with the index case-patient before being hospitalized. No pets, domestic animals, or birds were present in the immediate vicinity of the residences of either patient. However, the index case-patient worked daily as a butcher in an LPM near his residence during the 2 weeks before illness onset; the last known exposure in the LMP occurred on January 7 (Technical Appendix Figures 8,9). The second case-patient had no history of exposure to live birds or LPMs in the 2 weeks before his hospital admission. The 2 patients had no history of eating poultry or known contact with a person with febrile illness in the 2 weeks before onset.

The second case-patient (in bed 27) shared the same room and was in the adjacent bed to the index case-patient (in bed 26) during January 10–15 (Figure 2). During this period, the index case-patient remained mostly confined to his bed, whereas the second patient was ambulatory. Aerosol-generating procedures were performed for the index case-patient on January 14–15, including continuous oxygen mask inhalation and negative pressure suction. Endotracheal intubation and bag valve mask ventilation was used for 2 hours and mechanical ventilation for 5 hours until he was transferred to the ICU on January 15. During January 10–15, the index case-patient had continued high fever with sweating and was washed and had clothes changed by his wife. Neither patient had vomiting or diarrhea. The ward floor and surfaces were disinfected by using domiphenbromide (1:400 concentration) twice a day.

Three other patients shared the same ward during this period (Figure 2; Table 1). Bed 25 was occupied during January 2–17 by patient 3, a 78-year-old man with chronic B cell leukemia, and during January 17–22 by patient 5, a 61-year-old man with macroglobulinemia. After the index case-patient was transferred to the ICU, during January 15–23, bed 26 was occupied by patient 4, a 50-year-old man with acute myeloid leukemia (Figure 1). Patient 3 was H7N9 virus antibody–negative by HI test 42 days and 44 days after his most recent exposures to the second case-patient and index case-patient, respectively. Patients 4 and 5 were negative by rRT-PCR for influenza A viruses 1 day after their most recent exposures and were negative for H7N9 virus antibody by HI test 40 days after their most recent exposure to the second case-patient. Because the diagnosis of H7N9 virus infection was not suspected during their stay in this room, neither the index case-patient nor the second case-patient wore a protective mask, and the 25 staff or visitors who visited the room did not use any personal protective equipment. 

### Investigation of Contacts

A total of 68 close contacts of the index case-patient were identified, including 2 patients in the hematology ward, 7 patients in the ICU of the respiratory ward, 51 healthcare workers, 4 household members, and 4 social contacts. The median duration of exposure to the index case-patient was 3.5 hours (interquartile range [IQR] 2.5–6.5 hours]. No close contacts reported taking oseltamivir chemoprophylaxis. A total of 64 close contacts of the second case-patient were identified, including 3 patients in the hematology ward, 6 patients in the ICU, 51 healthcare workers, 2 household members, and 2 social contacts. None of the contacts developed acute respiratory symptoms. The median duration of exposure to at least 1 of the case-patients was 2 hours (IQR 1–3 hours) (Table 1).

Of the contacts of the index case-patient in the hematology ward, 31 close contacts (2 patients, 21 healthcare workers, 4 household members, and 4 social contacts) were traced. During the 7-day surveillance period, acute respiratory symptoms developed in 1 of 2 patients who shared the room in the hematology ward and in 2 of 4 social contacts. On January 23, a total of 8 throat swabs were collected from 2 of patients in the hematology ward, 4 household members, and 2 social contacts. Of these, only 1 (case-patient 2) was found to be infected with H7N9 virus. For patient 2, a total of 28 close contacts (3 patients, 21 healthcare workers, and 4 household members) were traced, and none of them had symptoms of disease. Three throat swab samples collected from the 3 patients were negative for H7N9 virus.

During January 15–19, a total of 27 close contacts of the index case-patient when he was in ICU (20 healthcare workers and 7 patients sharing the ICU ward) were traced; 6 of the patients and 4 of the healthcare workers had throat swab samples collected. One of symptomatic patients, a 71-year-old man who had been in the ICU during January 10–19 and had chronic obstructive pulmonary disease, pneumonia, and chronic renal failure, was positive for pH1N1 virus by rRT-PCR on throat swab samples collected on January 20. A total of 26 close contacts of the second case-patient (20 healthcare workers and 6 patients sharing the ICU ward) were traced. None of them none of them had signs or symptoms of disease. Throat swab samples were not collected.

Ten healthcare workers had contact with the index case-patient and 10 with the second case-patient for the periods these patients were in the infectious disease ward. All throat swab samples collected during January 20–24 from these 20 contacts were negative for H7N9 and pH1N1 viruses by rRT-PCR. Overall, serum samples from 84 contacts who consented to have serum collected for testing were negative for H7N9 virus antibodies by MN and HI assays.

On January 20, a total of 10 environmental samples from the LPM that the index case-patient worked in before his illness (Technical Appendix Figure 8) were collected; 1 of these was positive for H7N9 and H9N2 viruses, and another was positive for H7N9 virus. Two throat swab samples from workers in this LPM were negative for influenza A virus. Furthermore, throughout January 2014, environment surveillance results in Linhai District showed that 17 (60.71%) of 28 LMPs were positive for H7N9 virus (Technical Appendix Figure 9).

### Viral Genetic Sequence Analysis of H7N9 and pH1N1 Viruses

No virus isolate was obtained from the index case-patient, but the partial genome of the virus was obtained by direct sequencing from his pharyngeal swab specimens. Full-length membrane protein and nonstructural gene sequences and partial hemagglutinin and neuraminidase sequences were obtained. A virus isolate was obtained from the second case-patient, and the full virus genome was ascertained. Alignment of the membrane protein and neuraminidase sequences from the index case-patient with the H7N9 virus sequences from the virus isolated from the second case-patient showed that sequences for these 2 shared 99.5% and 100% nucleic acid sequence identity, respectively. Both case-patients had viruses that were oseltamivir-sensitive (E120V, H276Y and R294K in neuraminidase protein) but amantadine-resistant (S31N in matrix 2 protein). The pH1N1 virus neuraminidase gene segment from the index case-patient was 100% identical to that from the second case-patient.

### Serum Cytokine Levels

Serum levels of cytokines and chemokines in the index case-patient (10 days after illness onset) and the second case-patient (9 days after illness onset) were compared with those of 21 other patients infected with H7N9 virus. Levels of 13 cytokines and chemokines (granulocyte colony–stimulating factor, growth-regulated oncogene-α, interleukin [IL] 10, IL-12p40, IL-16, IL-18, IL-1 receptor antagonist, IL-2 receptor antagonist, IL-3, IL-6, IL-8, leukemia inhibitory factor, monocyte chemoattractant protein-1, monocyte chemoattractant protein 3, and stromal cell–derived factors) from the index case-patient and the second case- patient were higher than the upper bound of the 95% CIs of the mean levels of other patients infected with H7N9 virus (Table 2; Technical Appendix Figure 10). In contrast, levels of interferon-gamma (IFN-γ), IL-2, IL-12, and IL-4, which are correlated with adaptive immune responses to influenza viruses, were comparable to those observed in healthy persons but much lower than those observed in nonimmunocompromised H7N9 virus–infected patients (Technical Appendix Figure 11). The lack of these cytokines, which are associated with the development of T helper (Th) 1, Th2, and Th17 cells, might have led to an incapable adaptive immune response in these 2 case-patients and thereby enhanced the pathogenicity of the viruses.

**Table 2:** Cytokine and chemokine levels for controls compared with 2 patients with confirmed co-infection with avian influenza A(H7N9) and A(H1N1)pdm09 viruses, Taizhou Hospital, Zhejiang Province, China, January 2014*

Cytokine or chemokine	Healthy controls, n = 6			H7N9-infected controls. n = 21		Index case-patient	Case-patient 2	Change
	Mean	95% CI		Mean	95% CI			
G-CSF	27.6	21.9 to 33.3		47.5	32.1 to 62.9	3421.0	5,759.0	↑
GROa	260.2	79.6 to 440.8		164.8	89.4 to 240.2	320.8	757.4	↑
IL-10	0.8	0.4 to 1.2		17.8	12.9 to 22.7	42.2	234.1	↑
IL-12p40	719.8	59.5 to 1380.1		240.3	−28.2 to 508.8	5176.0	585.5	↑
IL-16	530.0	−71.7 to 1131.7		260.8	170.2 to 351.3	627.4	468.51	↑
IL-18	69.9	31.5 to 108.2		163.7	101.3 to 226.1	530.9	1,501.1	↑
IL-1Ra	111.8	89.5 to 134.1		218.3	164.3 to 272.2	506.7	1,340.1	↑
IL-2Ra	79.6	51.2 to 108.1		97.5	49.9 to 145.1	699.9	425.4	↑
IL-3	101.4	30.9 to 171.8		30.8	−0.8 to 62.4	815.3	210.4	↑
IL-6	4.1	2.8 to 5.4		91.5	50.7 to 132.3	819.6	22,009.0	↑
IL-8	7.0	2.7 to 11.4		62.2	45.5 to 79.0	1,228.3	2,075.3	↑
LIF	9.1	0.7 to 17.5		7.9	1.3 to 14.4	34.4	31.2	↑
MCP-1	7.0	2.9 to 11.0		238.4	91.8 to 384.9	569.7	757.4	↑
MCP-3	28.5	8.8 to 48.2		8.6	−1.4 to 18.7	28.8	39.5	↑
SDF-1a	83.0	43.4 to 122.6		72.6	37.3 to 107.9	641.8	273.7	↑
Extaxin	79.7	36.3 to 123.1		108.9	79.1 to 79.1	25.5	36.3	↓
GM-CSF	0.1	0.04 to 0.1		65.8	44.5 to 87.1	38.8	26.4	↓
IFN-γ	66.7	61.3 to 72.1		117.3	91.8 to 142.7	64.3	62.9	↓
IL-12 p70	8.0	5.3 to 10.7		32.7	25.7 to 39.7	18.0	6.0	↓
IL-2	3.9	1.9 to 5.8		17.8	13.2 to 22.4	5.3	7.9	↓
IL-4	2.9	2.2 to 3.5		8.4	6.5 to 10.4	1.2	1.7	↓
IL-5	1.7	1.1 to 2.2		7.2	4.5 to 10.0	2.1	1.8	↓
MIG	315.7	189.8 to 441.5		7,860.2	4245.5 to 11,474.9	2,246.4	429.8	↓
PDGF-bb	532.0	419.8 to 644.3		3,608.5	2,746.0 to 4,471.1	784.5	21.2	↓
RANTES	613.5	798.5 to 983.5		12,716.5	9,193.1 to 16,239.9	658.5	178.2	↓

*Levels are given in pg/mL. Arrows indicate cytokine and chemokine levels that were significantly higher (↑) or lower (↓) in the serum samples of the index patient and patient 2 compared with 95% CIs of the mean values of 21 other patients infected with the avian influenza A(H7N9) virus. G-CSF, granulocyte colony stimulating factor; GM-CSF, granulocyte macrophage colony–stimulating factor; GROa, growth-regulated oncogene-α; IFN-γ, interferon-gamma; IL, interleukin; MCP, monocyte chemoattractant protein; MIG, macrophage-induced gene ; PDGF-bb, platelet-derived growth factor bb; Ra, receptor antagonist; RANTES, regulated on activation normal T cell expressed and secreted; SDF, stromal cell–derived factors.

## Discussion

Although ≈600 cases caused by H7N9 virus have been reported, 41 clustered cases from 20 disease clusters have been reported. The clustered cases accounted for only 5.9% of the total cases, so there seems to be evidence of limited transmission (*10*). Our study found epidemiologic differences between 1 nosocomial cluster caused by co-infection with H7N9 and pH1N1 viruses and a previously documented family cluster induced by a single H7N9 virus.

The index case-patient had been exposed to an LPM that was retrospectively proven to be positive for influenza H7N9 virus. The second case-patient had no avian exposure but experienced onset of clinical disease within 4 days of close contact with the index case-patient. The second case-patient was co-infected with H7N9 and pH1N1 viruses, as was the index case-patient. Virus genetic sequences of H7N9 and pH1N1 viruses from the 2 case-patients were identical. These findings strongly suggest nosocomial human-to-human co-transmission of both influenza viruses in 2 non–blood-related patients with hematologic disorders. The findings also demonstrate a lack of transmission to other patients and healthcare workers, reinforcing the contention that H7N9 virus remains poorly transmissible from human to human. 

In this particular event, the incubation period was 4 days, compatible with previous estimates of incubation period (*11*). This incubation period was more plausible than the incubation periods of 13 days, 7 days, and 10 days reported for the Jiangsu (*3*), Shanghai, and Guangdong clusters, respectively (*12*,*13*), where infection from a common source cannot be ruled out. In other family clusters, the index patient’s illness was more severe, whereas most of the secondary cases were milder and rarely fatal (*14*,*15*). The greater disease severity in the second case-patient in our study might be attributable to the co-infection with influenza pH1N1 virus in combination with his underlying disease and the delayed initiation of antiviral therapy (*16*).

The exact route of transmission from the index case-patient to the second case-patient remains unclear. Transmission through respiratory droplets is possible because the index case-patient had repeated bouts of coughing, and he had several aerosol-generating procedures conducted during his hospitalization. Cross-infection through fomites, the unwashed hands of healthcare workers or visitors, or unsanitary medical equipment might also be possible. Before the 2 case-patients were confirmed to have H7N9 virus infection, no personal protective equipment was used. 

Serologic methods rarely yield an early diagnosis of influenza virus. Apart from their retrospective diagnostic value, serologic methods are applied in epidemiologic and immunologic studies. In our study, we conducted HI and MN tests for all contacts of the 2 case-patients under negative and positive quality control. However, we did not detect any serologic evidence of secondary transmission in approximately 84 contacts. The basic reproduction number (R_0_), a measure of transmission potential of H7N9 virus compared with pH1N1 virus, was 0.1 versus 1.7, indicating that the transmission potential of H7N9 virus was much lower than that of pH1N1 virus. Thus, prolonged exposure and the immunocompromised status of the 2 case-patients might have contributed to the transmission of infection. Co-infection of H7N9 and seasonal influenza A(H3N2) viruses has been reported in a patient from Jiangsu Province, China, but this patient recovered, possibly because of his young age (15 years) and early initiation of antiviral treatment; evidence of secondary transmission was not actively sought (*17*).

Patients with hematologic malignancy, such as chronic lymphocytic leukemia and polycythemia vera, have multiple immune defects (*18*–*21*), including defects in T-cell function and natural killer cell dysfunction, decreased antibody responses, and abnormal cytokine production (*21*). Patients with such disorders are thus more prone to new infections and prolonged virus shedding (*18*,*20*,*22*–*28*). For example, a patient with chronic lymphocytic leukemia who was not in an ICU was infected with pH1N1 virus and continued to shed virus for 59 days; antiviral resistance developed, consistent with the patient’s immunocompromised condition (*28*). In our study, the index case-patient was confirmed as H7N9 virus–positive on day 11 after illness onset, which suggests prolonged viral shedding in older, immunocompromised patients. However, the consequences of H7N9 patients with hematologic malignancies are poorly understood in terms of clinical outcomes and transmission potential.

Innate immune dysregulation might contribute to the pathogenesis of severe avian influenza (*29*). In our study, the 2 H7N9 virus–infected case-patients who died had chronic lymphocytic leukemia and polycythemia vera, respectively. Chronic lymphocytic leukemia is associated with dysfunction of innate and adaptive immunity (*21*,*30*,*31*). Cytokines, such as IL-8, associated with acute respiratory distress syndrome (*32*), and IL-6, associated with severe cytokine-release syndrome, can occur in chronic lymphocytic leukemia patients after treatment with rituximab, an anti-CD20 monoclonal antibody (*33*).The underlying disease (polycythemia vera) in the second case-patient is associated with Janus kinase 2 mutation V617F, which leads to constitutive tyrosine phosphorylation activity that promotes cytokine hypersensitivity, including IL-3 and stem cell factor (*34*,*35*). The abnormal signaling pathway in cells of patients with polycythemia vera can also contribute to growth of fibroblasts and microvascular endothelial cells and induce the production of profibrogenic and angiogenic cytokines (*36*).

Hypercytokinemia has been reported in the peripheral blood of H7N9 virus–infected patients with substantially elevated levels of cytokines and chemokines (e.g., IL-1β, IL-6, IL-8, IL-10, IL-12, IL-17, monokine induced by IFN-γ, macrophage inflammatory proteins 1α and 1β, monocyte chemotactic protein 1, interferon-induced protein of 10 kDa, and regulated on activation normal T cell expressed and secreted [RANTES]) (*5*,*29*,*37*,*38*). Despite underlying immune defects in the context of steroid therapy, the 2 H7N9 virus–infected case-patients in our study mounted strong proinflammatory cytokine responses (e.g., production of IL6, IL8, and monocyte chemotactic protein 1), even stronger than those observed in other nonimmunocompromised patients with H7N9 infection. On the other hand, levels of cytokines known to correlate with immune protection against influenza viruses, such as IL-2, IL-4 and IFN-γ, which contribute to development of Th1, Th2, and Th17 cells, were observed to be much lower in the 2 case-patients reported here compared with other H7N9 virus–infected patients. Thus, disorders of innate immune and adaptive immune responses in these 2 case-patients might have contributed to the disease severity and fatal outcomes.

In summary, our findings strongly suggests nosocomial human-to-human co-transmission of H7N9 and pH1N1 viruses between 2 immunocompromised case-patients with hematologic diseases, with no evidence of transmission to others. The deaths of these 2 case-patients might have been attributable to co-infection with pH1N1 virus, delayed initiation of antiviral therapy, and the patients’ immunocompromised status. These findings suggest the need for awareness and early testing for influenza in hematology units and for liberal use of early antiviral treatment if patients exhibit ILI symptoms. Implementing rigorous infection control practices might minimize cross-transmission. Avoiding the use of corticosteroids in patients with infection also needs to be emphasized.

Technical Appendix Additional figures detailing hospital setting, clinical courses, and laboratory findings of 5 patients who shared the same hematology ward, Taizhou Hospital, Zhejiang Province, China, January 10–15, 2014.
