# Supplementary material for: Nosocomial Co-Transmission of Avian Influenza A(H7N9) and A(H1N1)pdm09 Viruses between 2 Patients with Hematologic Disorders
Source: Emerg Infect Dis. 2016 Apr;22(4):598–607. doi: 10.3201/eid2204.151561 (PMC4806937; doi:10.3201/eid2204.151561)
Supplement: Technical Appendix — Additional figures detailing hospital setting, clinical courses, and laboratory findings of 5 patients who shared the same hematology ward, Taizhou Hospital, Zhejiang Province, China, January 10–15, 2014. [file 15-1561-Techapp-s1.pdf]

Article DOI: http: //dx.doi.org/10.3201/eid2204.151561

Nosocomial Co-Transmission of Avian Influenza A(H7N9) and A(H1N1)pdm09 Viruses between 2 Patients with Hematologic Disorders

Technical Appendix

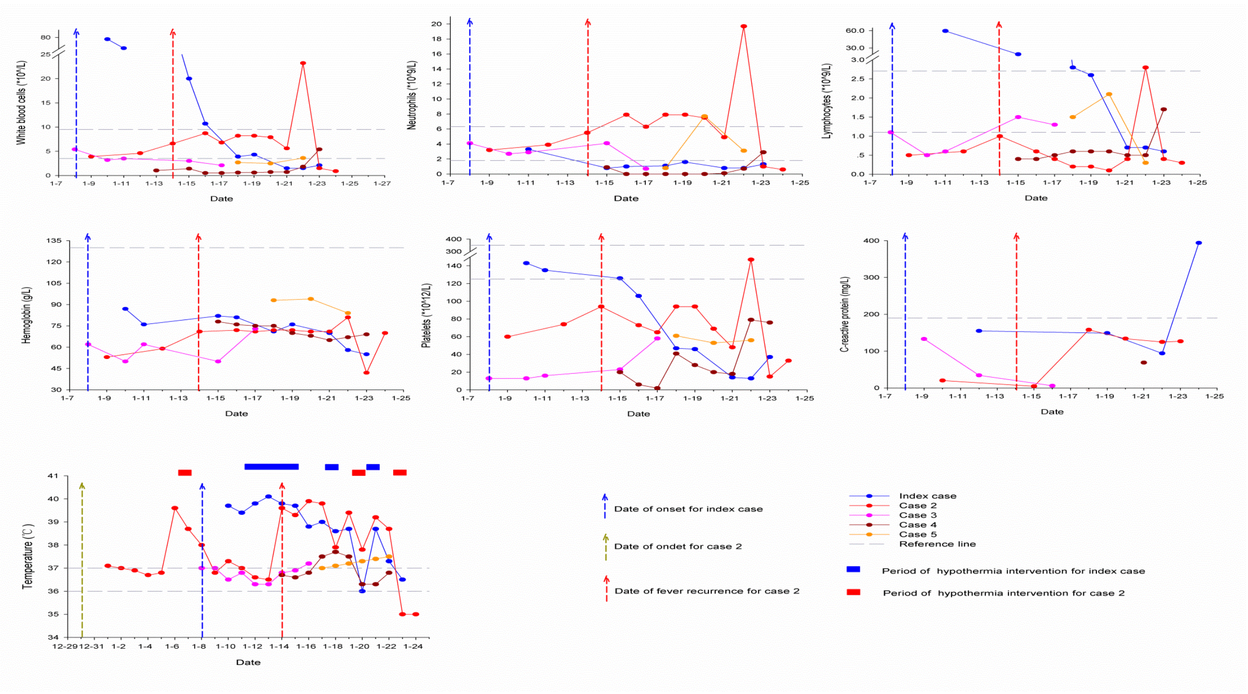

Technical Appendix Figure 1. Progress of clinical symptoms and biochemical indices of index patient and patient 2 with co-infection with avian influenza A(H7N9) and A(H1N1)pdm2009 viruses and 3 non–H7N9-infected patients who shared the same hematology ward, Taizhou Hospital, Zhejiang Province, China, January 2014. Reference ranges of the indicators are white blood cells 3.5–9.5 ($\times 10^9$/L); neutrophils 1.8–6.3 ($\times 10^9$/L); lymphocytes 1.1–3.2 ($\times 10^9$/L); hemoglobin 130–175 (g/L); platelets 125–350 ($\times 10^{12}$/L); C-reactive protein <190 (mg/L); temperature 36.5–37.0°C. Dates of illness onset for index patient and patient 2 were January 8 and January 14, 2014, respectively.

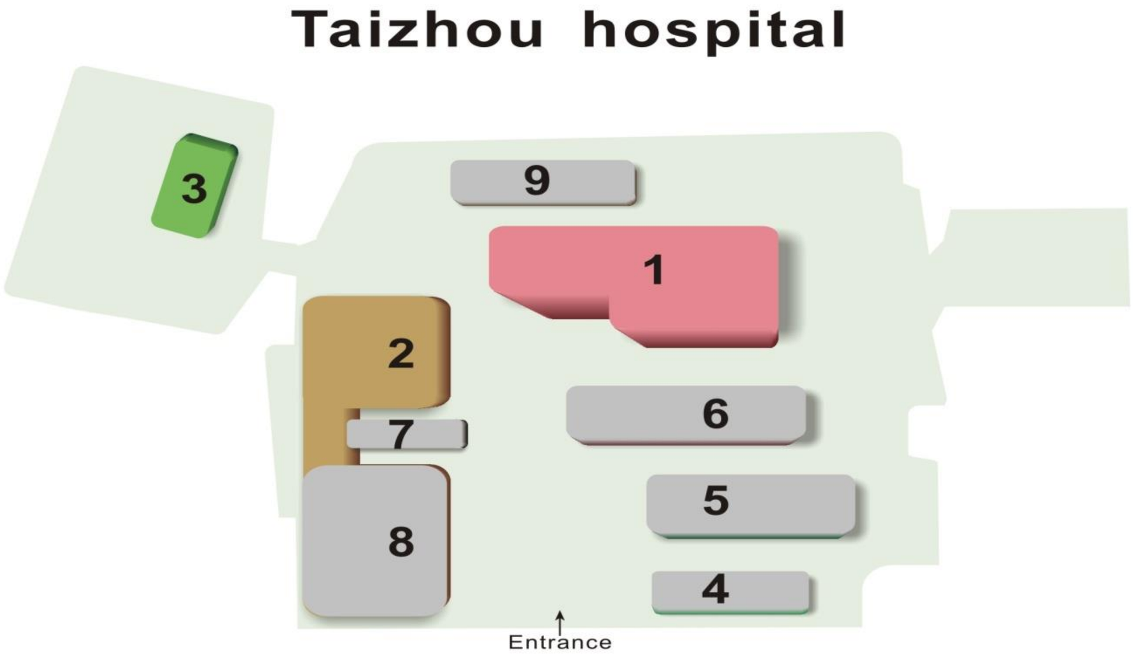

Technical Appendix Figure 2. Schematic diagram of Taizhou Hospital, Zhejiang Province, China, January 2014. 1. Inpatient building 1 (2F: intensive care unit [ICU], 19F: hematology department); 2. Inpatient building 2 (2F: ICU of respiratory infections ward); 3. Inpatient building 3 (entire floor is for infectious diseases department); 4. Auxiliary building 1; 5. Outpatient building; 6. Emergency building; 7. Administrative building; 8. Inspection and testing center; 9. Auxiliary building 2.

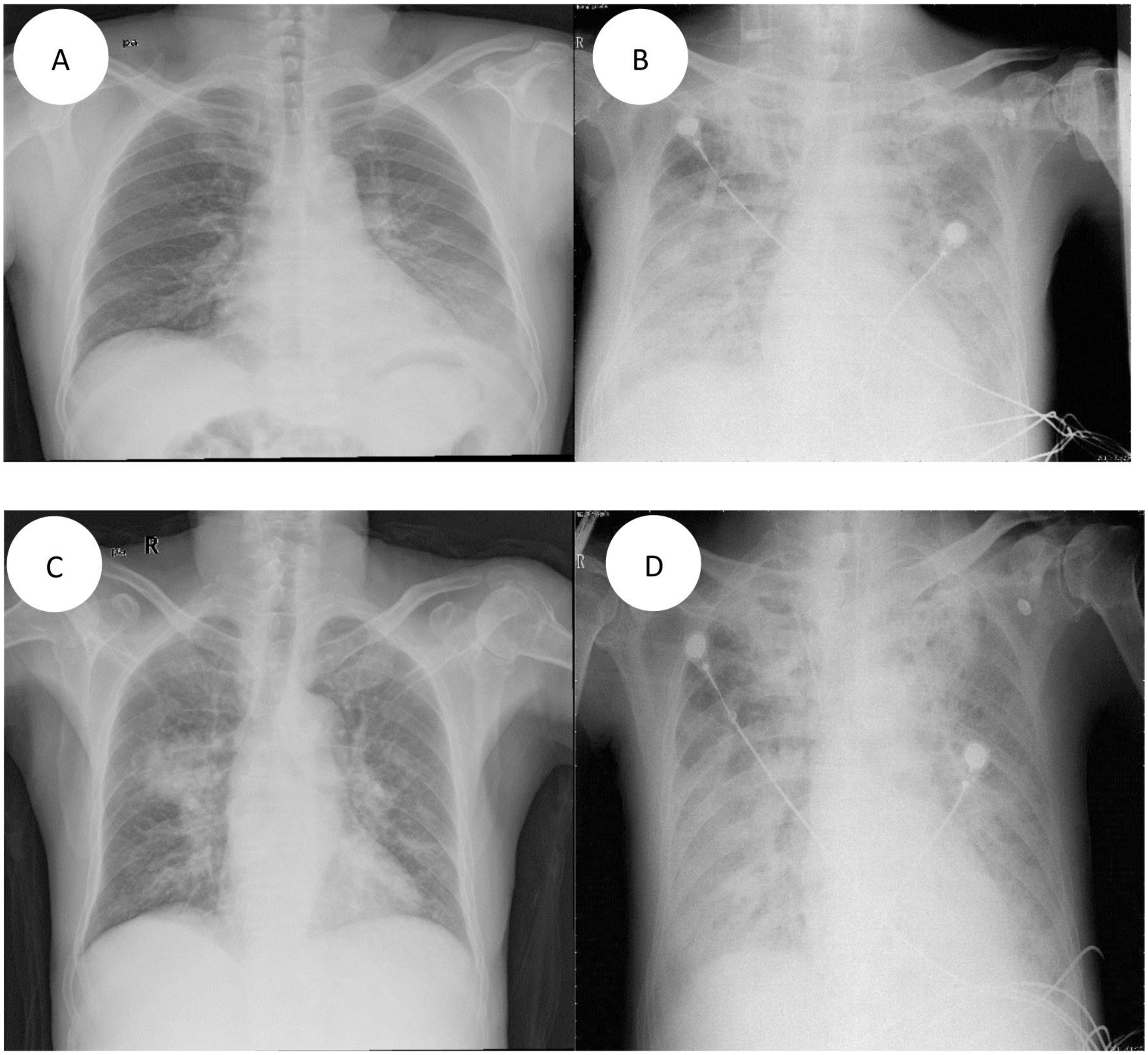

Technical Appendix Figure 3. Chest radiographs of the chest of index patient and patient 2 with co-infection with avian influenza A(H7N9) and A(H1N1)pdm09 viruses, Taizhou Hospital, Zhejiang Province, China, January 2014. A) Chest radiograph obtained on January 11 showed consolidation of the lower left lung lobe. B) On January 23, ground-glass opacity and consolidation could also be seen in the 2 upper and lower left lobes along with neck and chest subcutaneous pneumatics, and bilateral pleural effusion. C) On January 15, the ground-glass opacities were found in bilateral lower lobes. D) On January 22, ground-glass opacities and consolidation were identified in bilateral upper and lower lobes.

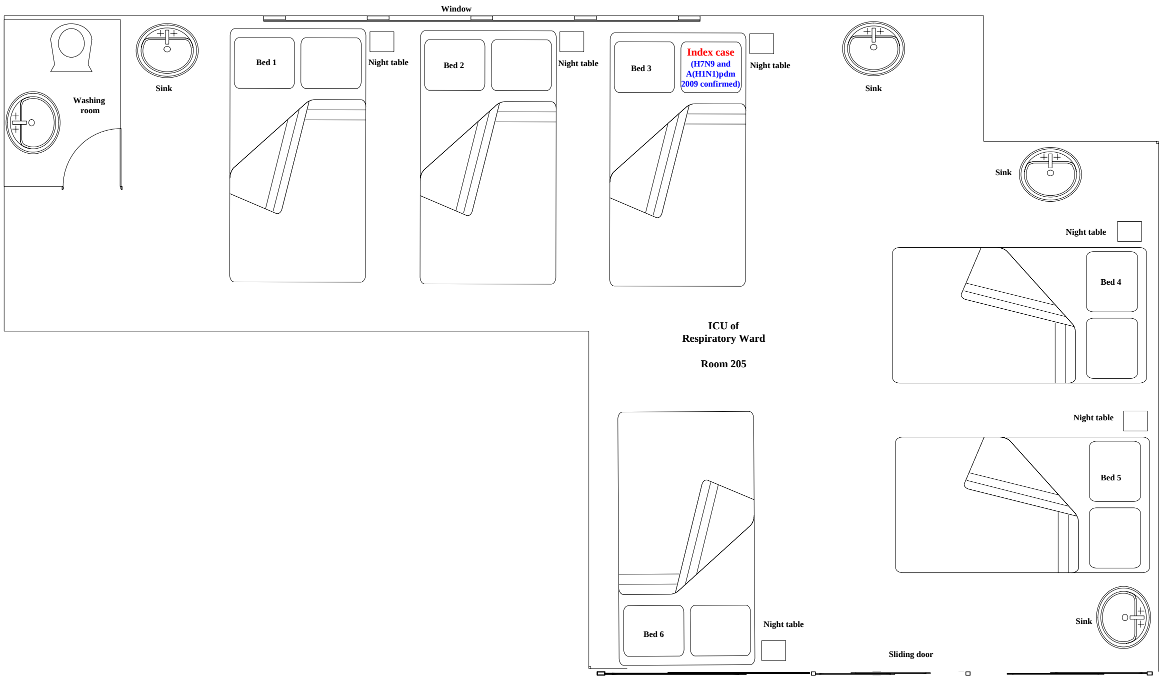

Technical Appendix Figure 4. Floor plan of the intensive care unit of respiratory ward where index patient with avian influenza A(H7N9) and A(H1N1)pdm2009 virus co-infection and the 3 non–H7N9-infected patients were housed during January 15–19, 2014, at Taizhou Hospital, Zhejiang Province, China.

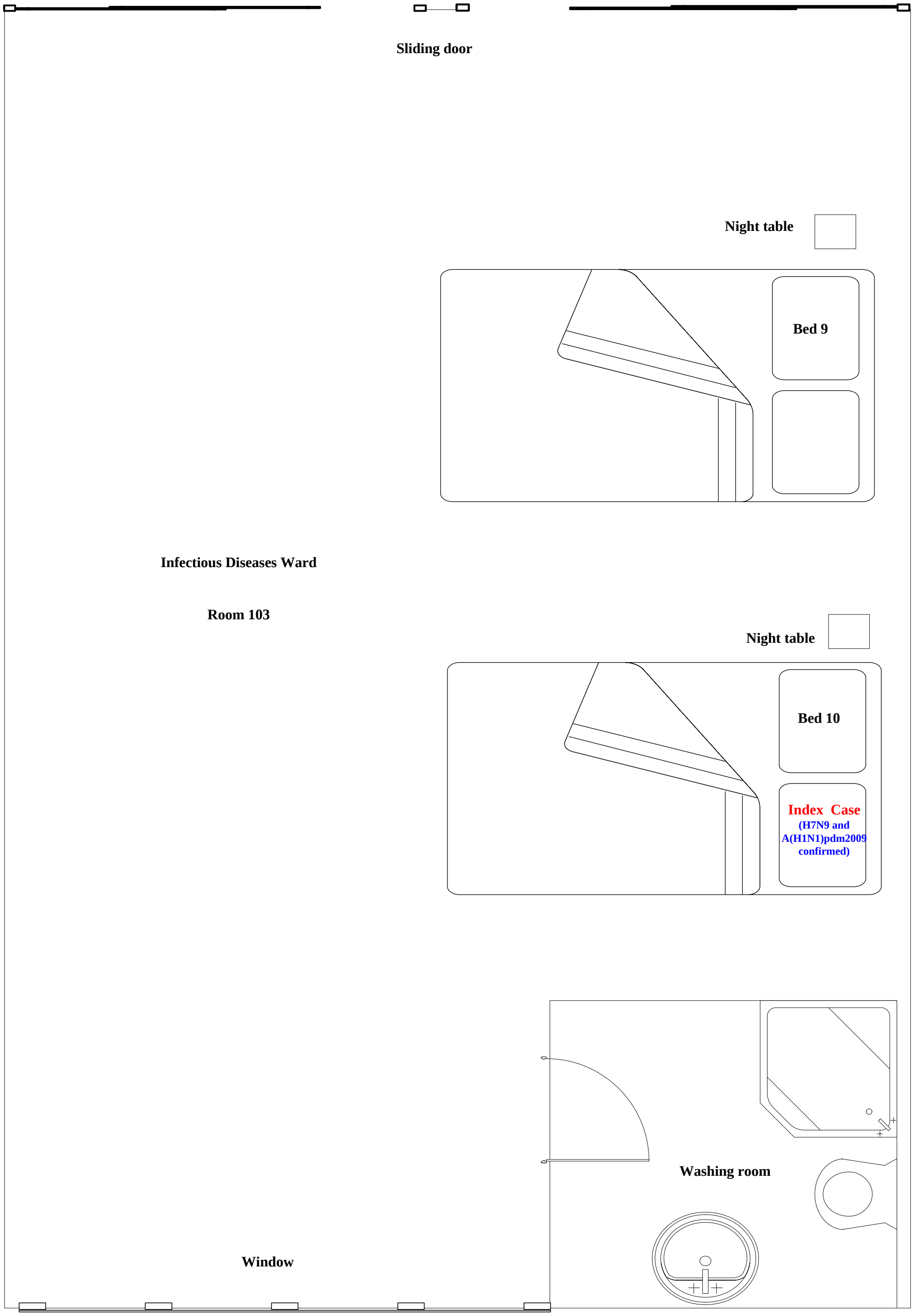

Technical Appendix Figure 5. Floor plan of the infectious diseases ward where index patient confirmed as having avian influenza A(H7N9) and A(H1N1)pdm2009 virus co-infection was isolated during January 19–23, 2014, at Taizhou Hospital, Zhejiang Province, China.

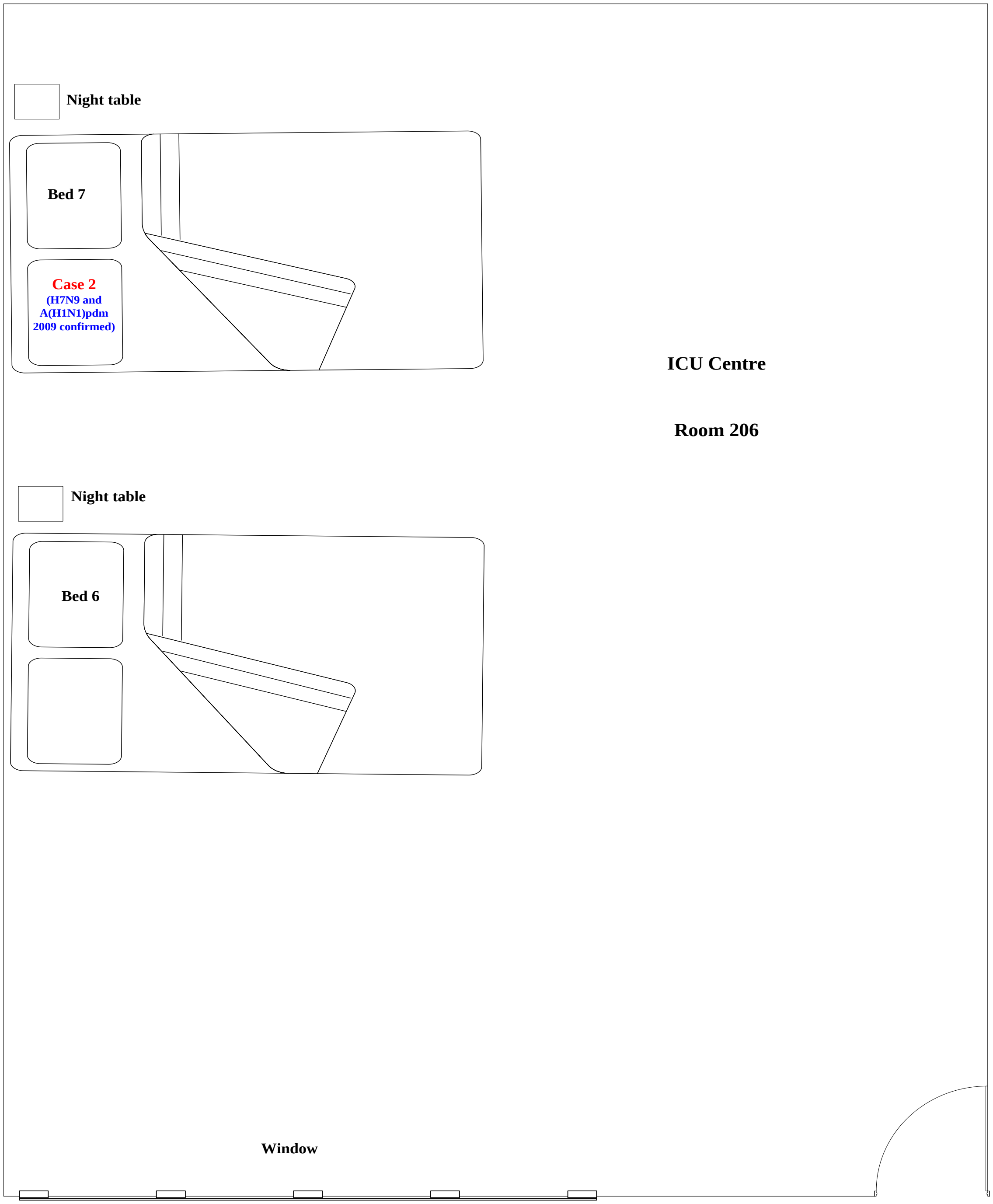

Technical Appendix Figure 6. Floor plan of the intensive care unit where patient 2 confirmed as having avian influenza A(H7N9) and A(H1N1)pdm2009 virus co-infection and 3 H7N9-negative patients lived together during January 19–21, 2014, at Taizhou Hospital, Zhejiang Province, China.

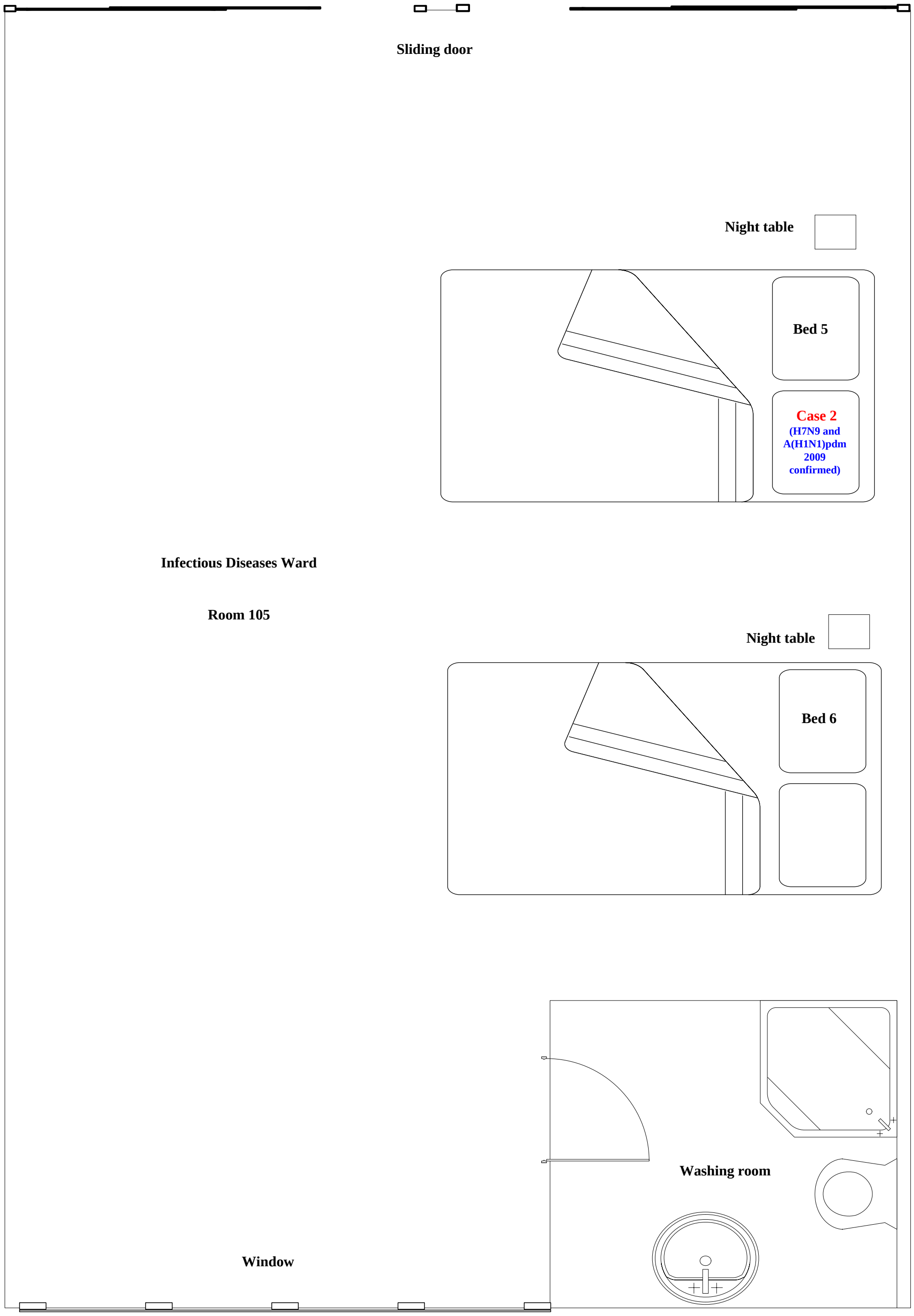

Technical Appendix Figure 7. Floor plan of the infectious diseases ward where patient 2 confirmed as having avian influenza A(H7N9) and A(H1N1)pdm2009 virus co-infection was isolated during January 21–24, 2014, at Taizhou Hospital, Zhejiang Province, China.

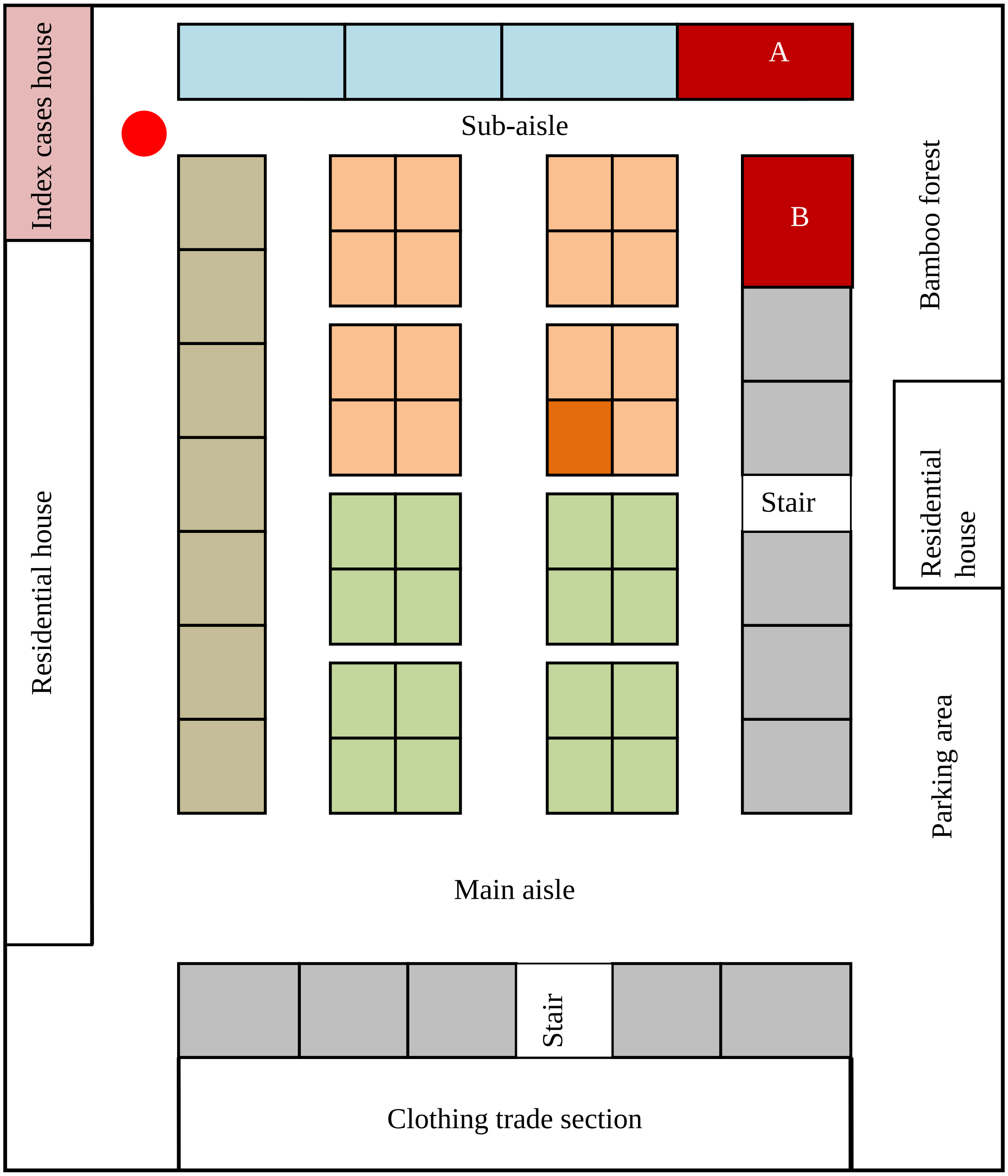

Technical Appendix Figure 8. Floor plan of live poultry market in Linhai District in Taizhou, Zhejiang Province, China, where the index patient sold meat 2 weeks before illness onset on January 8, 2014. Stall B tested positive for avian influenza A(H7N9) virus, whereas stall A tested negative by real-time reverse transcription PCR conducted on environmental samples collected on January 20, 2014.

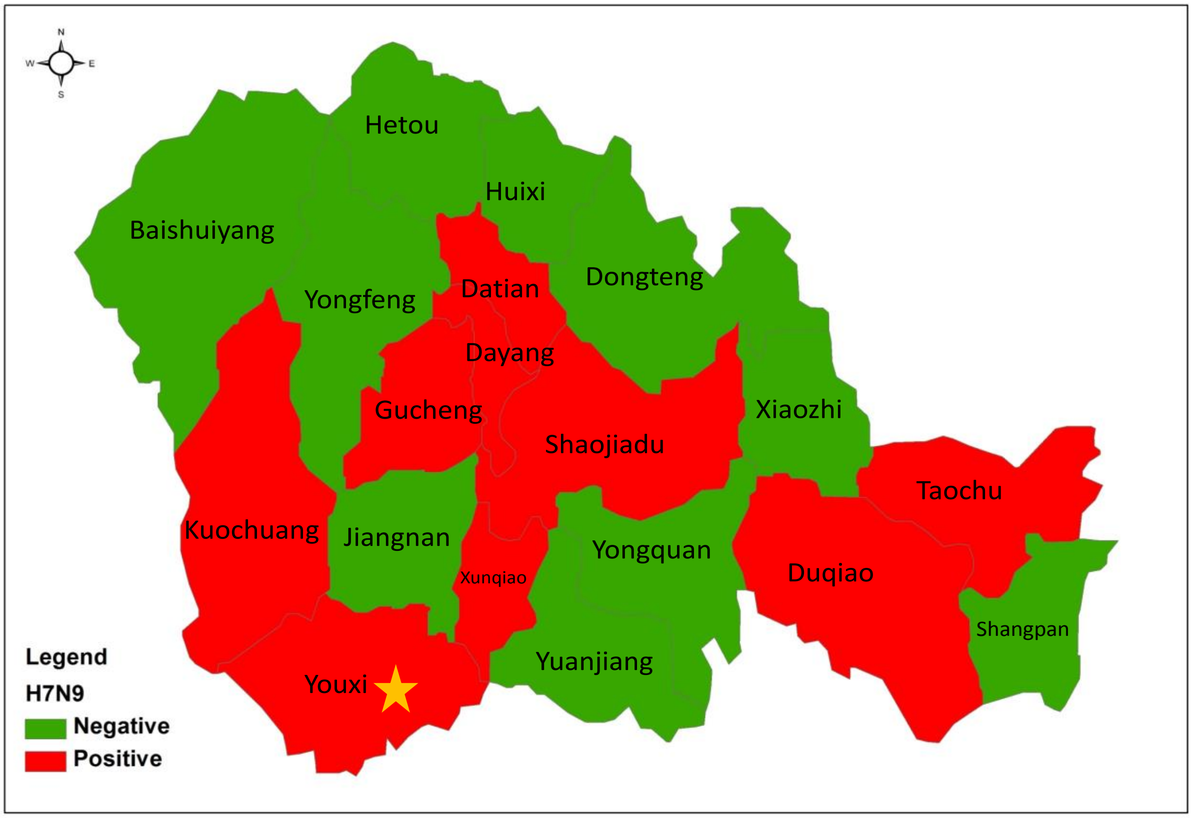

Technical Appendix Figure 9. Environmental surveillance for avian influenza A(H7N9) virus was performed by using real-time reverse transcription PCR in 15 towns and 28 live poultry markets of Linhai District in Taizhou, Zhejiang Province, China, during January 2014. Red color indicates H7N9 virus–positive towns; green color indicates H7N9 virus–negative towns. The index patient had worked in a live poultry market.

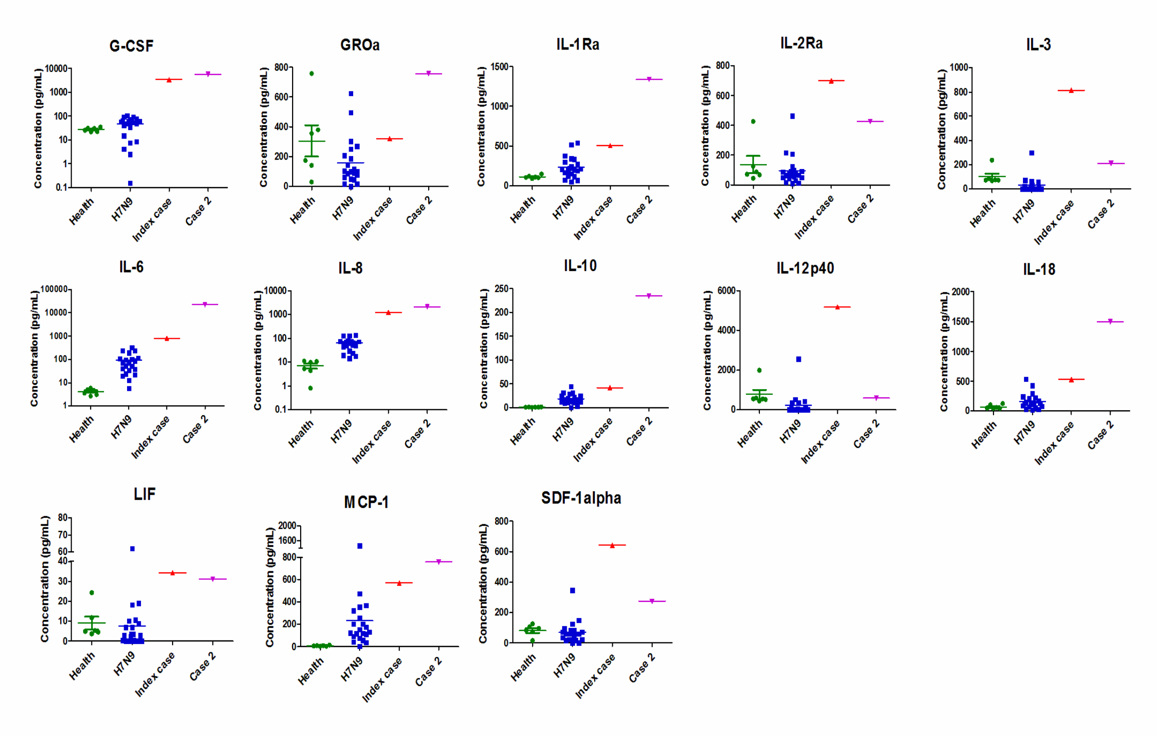

Technical Appendix Figure 10. Cytokines that are increased in 2 patients with confirmed avian influenza A(H7N9) and A(H1N1)pdm09 virus co-infection, Taizhou Hospital, Zhejiang Province, China, January 2014. Serum samples from the index patient and patient 2 were collected on day 10 and day 9 of illness, respectively. Stored serum samples from 21 other H7N9 virus–infected patients without immunocompromising conditions and 6 healthy controls are also shown with 95% CIs. Levels of chemokines and cytokines were measured. GSF, granulocyte colony–stimulating factor; GROa, growth-regulated oncogene-α; H7N9, avian influenza A(H7N9) virus; IL, interleukin; LIF, leukemia inhibitory factor; MCP, monocyte chemoattractant protein; Ra, receptor antagonist; SDF-1a, stromal cell–derived factor-1a.

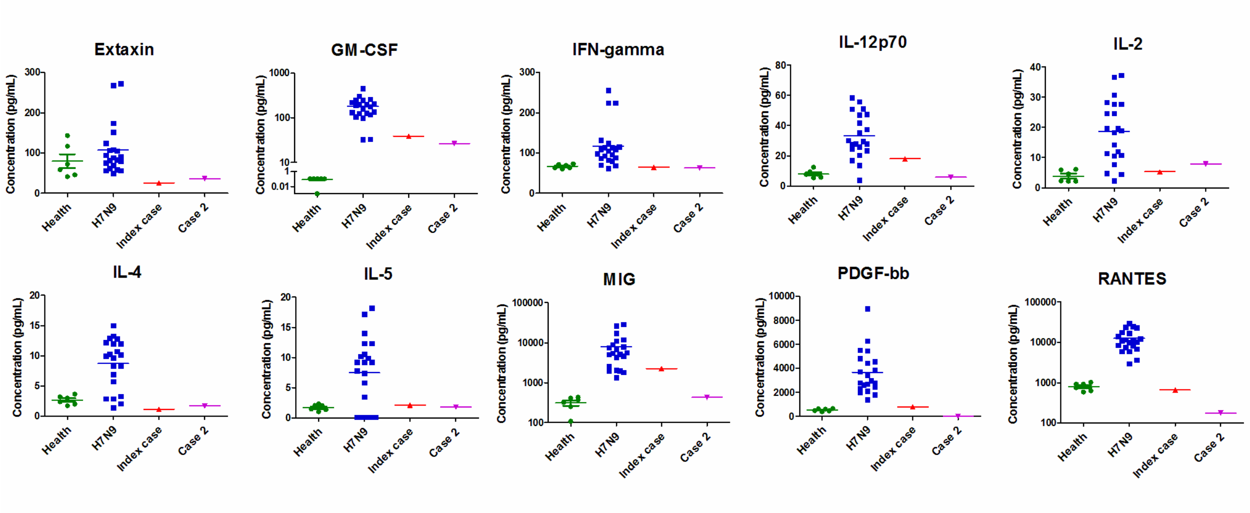

Technical Appendix Figure 11. Cytokines and chemokines levels in index patient and patient 2 compared with patients with avian influenza A(H7N9) virus infection, Taizhou Hospital, Zhejiang Province, China, January 2014. Levels of cytokines and chemokines that were much lower than those observed in nonimmunocompromised H7N9 virus–infected patients are presented. GM-CSF, granulocyte macrophage colony stimulating factor; IL, interleukin; MIG, monokine induced by interferon-gamma; PDGF-bb, platelet-derived growth factor bb; RANTES, regulated on activation normal T cell expressed and secreted.
